# *RyR2* QQ2958 Genotype and Risk of Malignant Ventricular Arrhythmias

**DOI:** 10.1155/2016/2868604

**Published:** 2016-01-20

**Authors:** Francesca Galati, Antonio Galati, Serafina Massari

**Affiliations:** ^1^Department of Biological and Environmental Sciences and Technologies, University of Salento, 73100 Lecce, Italy; ^2^Department of Cardiology, “Card. G. Panico” Hospital, Tricase, 73039 Lecce, Italy

## Abstract

Ventricular arrhythmias are one of the most common causes of death in developed countries. The use of implantable cardiac defibrillators is the most effective treatment to prevent sudden cardiac death. To date, the ejection fraction is the only approved clinical variable used to determine suitability for defibrillator placement in subjects with heart failure. The purpose of this study was to assess whether genetic polymorphisms found in the ryanodine receptor type 2 (Q2958R) and histidine-rich calcium-binding protein (S96A) might serve as markers for arrhythmias. Genotyping was performed in 235 patients treated with defibrillator for primary and secondary prevention of arrhythmias. No significant association was found between the S96A polymorphism and arrhythmia onset, whereas the QQ2958 genotype in the ryanodine receptor gene was correlated with an increased risk of life-threatening arrhythmias. Concurrent stressor conditions, such as hypertension, seem to increase this effect. Our findings might help to better identify patients who could benefit from defibrillator implantation.

## 1. Introduction

Sudden cardiac death (SCD) is one of the most frequent causes of death in industrialized countries. SCD is commonly the result of ventricular tachycardia (VT) and/or ventricular fibrillation (VF) that occur secondary to a complex interplay between a susceptible myocardial substrate typically affected by cardiomyopathy and a transient trigger. The use of implantable cardioverter-defibrillator (ICD) is the most effective treatment to prevent this disease, because it may terminate the arrhythmia by low-voltage antitachycardia pacing or high-energy cardioversion. However, only a minority of patients benefit from these devices, because the majority of patients with an ICD have never received a shock appropriate for VT or VF. Furthermore, a substantial number of patients, who die suddenly, are not identified as high risk prior to death and do not receive an ICD implantation [1]. Although numerous clinical and serum biomarkers have been investigated for use in the risk stratification of SCD [2], the ejection fraction (EF) remains the only approved clinical variable that is used to determine suitability for ICD placement in subjects with heart failure (HF). As a result, there is a substantial interest in identifying more reliable predictors that could help to discriminate which patients are most likely to benefit from an ICD implant. The identification of genetic alterations responsible for rare hereditary arrhythmic diseases, such as Brugada syndrome, long QT, and catecholaminergic polymorphic ventricular tachycardia (CPVT), has focused the attention on the molecular basis of arrhythmias, particularly on the role of the calcium channels and associated proteins. The ryanodine receptor type 2 (RyR2), which is expressed primarily in cardiac muscle [3], is one of the three isoforms of the family of ryanodine receptors that regulate the duration and amplitude of the Ca^2+^ flow from the sarcoplasmic reticulum (SR). It is well known that aberrant diastolic Ca^2+^ release via RyR2 leads to contractile dysfunction by reducing the SR Ca^2+^ content. This provides a substrate for delayed after depolarisation (DAD), which ultimately leads to lethal arrhythmias [4]. Recently, Ran et al. [5] found that the G1886S variant (rs3766871) of the* RyR2* gene was associated with an increased risk of ventricular arrhythmias and sudden cardiac death. The histidine-rich Ca-binding protein (HRC), expressed predominantly in striated muscle, is another SR component involved in the regulation of the Ca^2+^ uptake [6, 7], accumulation [8], and release from the sarcoplasmic reticulum [7, 9]. As reported in previous studies [10–12], ion channels polymorphisms have the potential to modify the clinical phenotype. These findings suggested the idea that also polymorphisms in RyR2 and HRC genes might have the same potential, thus representing an important factor in determining the risk of arrhythmia in HF patients who could benefit from an ICD implantation. The most common RyR2 polymorphism is RyR2-Q2958R (rs34967813, A/G), described for the first time by Tiso et al. [13], with a heterozygous prevalence of 34% in Caucasians and 10% in African Americans. It is localized within the area of interaction with the RyR2 modulator [14] and has remained highly conserved during the evolution of the ryanodine receptors [14–17]. Therefore, it will be of interest to study the functional consequences of this variation. HRC is known as an effective regulator of RyR2 activity and SR Ca^2+^ release. A genetic variant of HRC, Ser96Ala (rs3745297, G/T), disrupts the Ca^2+^ microdomain around the RyR2, as it alters the Ca^2+^ dependent association of RyR2 and HRC [18] and may enhance RyR2 activity from the SR luminal side, increasing uncontrolled Ca^2+^ release and induced Ca^2+^ instability. Arvanitis et al. [19] identified an association between HRC-S96A and malignant ventricular arrhythmias in patients with idiopathic dilated cardiomyopathy. In this study, we investigated whether these two polymorphisms (RyR2-Q2958R and HRC-S96A) are associated with the occurrence of spontaneous ventricular arrhythmias in patients receiving an ICD for primary and secondary prevention of SCD.

## 2. Materials and Methods

### 2.1. Patients and Procedures

We enrolled 235 unrelated Caucasian patients, from Salento (Southern Italy), who were consecutively admitted between January 2009 and September 2012 at the Department of Cardiology of Hospital “Card. G. Panico,” and treated with an ICD, according to class I or class II indications of ACC/AHA/HRS guidelines [20, 21]. Patients with long QT syndrome, Brugada syndrome, or CPVT were excluded from the study. 157 subjects (66.8%) had an ICD implantation for primary prevention and 78 patients (33.2%) for secondary prevention. Reversible causes of ventricular arrhythmias, such as acute ischemia, electrolyte abnormalities, and QT prolonging medication use, were ruled out. During a mean follow-up time of 44 ± 13 months, 23 patients of the 157 experienced at least one episode of ventricular arrhythmia and they formed group I with the 78 patients of the secondary prevention. The remaining 134 patients of the 157, who did not develop ventricular arrhythmias, formed group II. Ventricular tachycardia was defined by the following characteristics: (i) a regular wide QRS complex (>120 milliseconds) tachycardia at a rate of more than 100 beats per minute and with a uniform and stable QRS morphology of the consecutive beats and (ii) the arrhythmia lasting ≥30 seconds or causing hemodynamic collapse in <30 seconds.

Demographic, clinical, and routine laboratory data were collected from all patients using a structured data form. Before implantation, a transthoracic echocardiogram and a coronary angiography were performed for all patients.

The presence of ischemic heart disease was defined as prior myocardial infarction and/or angina with hospitalization and/or an infarct and/or major ischemia patterns on electrocardiogram or angiographically documented coronary artery disease (>50% stenosis in ≥1 coronary artery). Dilated cardiomyopathy was defined as enlargement of the heart cavity and systolic dysfunction of one or both ventricles in the absence of congenital, coronary, hypertensive, valvular, or pericardial heart disease. The diagnostic criteria included the identification of an ejection fraction (EF) ≤35% and/or a fractional shortening <25%, in association with a left ventricular (LV) end-diastolic dimension >112% of the predicted value corrected for age and body surface area. LV ejection fraction was categorized as ≤35% or >35% according to the ACC/AHA/HRS guidelines [20] for ICD implantation. Patients were divided into the four NYHA (New York Heart Association) classes, based on how much they were limited during physical activity.

Hypertension was defined as a systolic blood pressure ≥140 mmHg or a diastolic blood pressure ≥90 mmHg or the use of antihypertensive medications. Diabetes was defined as a fasting plasma glucose level >7.0 mmol/L or a nonfasting plasma glucose level >11.1 mmol/L or the use of antidiabetic medications. Dyslipidemia was defined as elevated total (>240 mg/dL) or low-density lipoprotein (LDL >130 mg/dL) cholesterol levels or low levels of high-density lipoprotein cholesterol (HDL <40 mg/dL) or elevated triglycerides levels (>150 mg/dL). Smoking was categorized as nonsmoking or current smoking (currently smoking or stopped <1 year ago).

All patients had an ICD with a VT and VF programming which allowed the analysis of stored intracardiac electrograms and/or RR-intervals of ventricular tachyarrhythmias with a cycle length (CL) ≤330 ms. ICDs employed a stepwise analysis of morphology, rate, stability, atrioventricular association, and onset (ventricular acceleration, atrial acceleration or nonacceleration). All patients were followed up in our outpatient clinic at six-month intervals.

100 healthy subjects from the same population were also enrolled. We ruled out any disease by studying patients' medical history, by performing a physical examination, or by performing an echocardiogram and a chest radiograph.

The study was approved by the local ethics committee and conducted in accordance with the guidelines of the declaration of Helsinki. Informed consent was obtained from all subjects prior to participation.

### 2.2. DNA Analysis

DNA extraction was carried out on total blood using Archive Pure DNA Blood Kit (5-PRIME, Hamburg, Germany) according to the manufacturer's recommended protocol.

We genotyped our patients for the following two variants:

- *RyR2* Q2958R (rs34967813, A/G), involving a substitution of adenine with guanine within exon 61, which results in the substitution of arginine for glutamine;
- *HRC* S96A (rs3745297, G/T), involving a substitution of guanine with thymine within exon 1, which results in the substitution of alanine for serine.

The DNA polymorphism analyses were performed using AS-PCR. The primers were designed with the Primer3 software [22]. Primer sequences, annealing temperature, and amplification product sizes are shown in Table 1. PCR amplifications were carried out in a total reaction volume of 25 *μ*L, with each reaction containing 100 ng of gDNA, 5 pmol of each primer, 10 mM dNTPs, 2.5 U Taq 5-Prime Eppendorf (5-PRIME, Hamburg, Germany), and 1x reaction buffer. The reaction cycle conditions consisted of an initial denaturation step at 94°C for 5 min, followed by 35 cycles of 30 s denaturation at 94°C, 30 s annealing at varying temperatures (see Table 1 for specific annealing temperatures), and 30 s extension at 68°C, with a final extension at 68°C for 5 min.

Allele-specific primers were constructed by introducing a one-base mismatch sequence before the SNP site. After agarose-gel electrophoresis, the PCR product was visualized with ethidium bromide, photographed, and genotyped.

Due to deviation from Hardy-Weinberg equilibrium (HWE), to exclude genotyping errors, all genomic DNAs genotyped for* RyR2* Q2958R were subjected to direct sequencing. Primers used for direct sequencing were CTACAGATGGTGGCAGCAGA (upper primer) and GCAGGACTAAGGTCCCACAA (lower primer).

### 2.3. Statistical Analysis

Continuous data are expressed as the mean ± standard deviation; categorical data are expressed as a percentage. A goodness of fit test for normality and a Brown-Forsythe or Levene test for homogeneity of variances were used to assess the applicability of parametric tests. Differences between mean data were compared by Student's *t*-test for the normally distributed continuous variables or by the Mann-Whitney test for nonnormally distributed variables. Differences in genotype frequencies and other categorical data between cases and controls were compared with Fisher's exact test (mid-*p* exact *p* value) and, in Hardy-Weinberg disequilibrium (HWD), with Armitage trend test. The consistency of the genotype frequencies with the HWE was tested using a chi-squared goodness-of-fit test on a contingency table of observed versus expected genotypic frequencies in cases and controls. Post hoc evaluations, where necessary, were performed by means of the Bonferroni correction. The MedCalc Statistical Software version 13.3 (MedCalc Software bvba, Ostend, Belgium; http://www.medcalc.org; 2014) was used for the multivariate logistic regression analysis. A two-sided *p* value <0.05 was considered significant for all tests.

## 3. Results

Table 2 provides a summary of the characteristics of our study population. Overall, 83 (35.3%) patients had idiopathic dilated cardiomyopathy (IDCM), 108 (46.0%) patients had dilated ischemic heart disease (IHD), 29 (12.3%) patients exhibited nondilated IHD, and 15 (6.4%) patients had other heart diseases (HDs). Of the total patients in our study, simultaneous cardiac resynchronization therapy was used in 64 (27.2%) patients, 18 (17.8%) in group I and 46 (34.3%) in group II.

Although more male patients were included in group I than in group II, no other significant differences were observed between the two groups with regard to demographic data. With respect to HF aetiology, the difference in the distribution of cardiac pathologies was due to the different ACC/AHA/HRS guidelines indications (in nondilated IHD, an ICD is implanted only for secondary prevention, while in dilated cardiomyopathy, it is indicated in primary prevention). Comparable values for the clinical (NYHA classification) and echocardiographic (EF and ventricular size) prognostic markers were obtained in both groups. Over 45.5% of the total population was hypertensive, 38.3% was diabetic and 40.8% was dyslipidemic; however, hypertension, diabetes, and dyslipidemia were distributed in a uniform manner between the two groups (49.5% versus 42.5%, 39.6% versus 37.3%, and 41.6% versus 40.3%, resp.). Atrial fibrillation was present in 19.1% of our patients and was equally distributed between the two groups (14.9% versus 22.4%; *p* = 0.1807). No significant difference in pharmacological treatment was observed between the groups, with the exception of the antiarrhythmic drug amiodarone, which was taken by 60.4% of patients with VT/VF versus 25.4% of patients in the other group, because it was always given after the first episode of VT/VF documented by the ICD to prevent further arrhythmic episodes and ICD discharge.

No patient was lost to follow-up, during which 2 patients (2.0%) in group I and 2 patients (1.5%) in group II died, all for refractory heart failure. 11 patients (10.9%) in group I and 26 patients (19.4%) in group II were hospitalized for heart failure (*p* = 0.1028).

The genotypic distribution of the two polymorphisms in the overall cohort and with respect to arrhythmia occurrence is reported in Table 3.

The genotype distribution of the* HRC* S96A polymorphism is in HWE in the total population as in the two study groups. Conversely, the genotype distribution of the* RyR2* Q2958R variant showed deviation from HWE with a high percentage of heterozygotes in the total population and in the two groups.

However, the* RyR2* polymorphism genotypic distribution was not significantly different than that of a control population of 100 healthy subjects recruited within the same medical centre (4% QQ, 94% QR, and RR 2%).

When we compared our group of 101 subjects with ventricular arrhythmias (group I) to 134 patients without documented arrhythmias (group II), we found no significant difference in the distribution of the* HRC* S96A genotypes between them. Conversely, the distribution of* RyR2* Q2958R genotypes was significantly different between the two groups (*p* = 0.0040 with Fisher's exact test and *p* = 0.0086 using Armitage trend test), with a higher percentage of the QQ genotype in group I (13.9%) compared to group II (3.0%). A post hoc analysis with Bonferroni's correction for pairwise comparisons confirmed the significant difference. According to these data, the subjects with the QQ genotype seem to be more susceptible to VT/VF development (RR 1.95; IC 95% 1.45–2.62; *p* = 0.0018).

Multiple logistic regression analysis revealed that the correlation between the QQ genotype and the risk of VT/VF (OR 2.5559; 1.1394 to 5.7334; *p* = 0.0228) is independent from other clinical characteristics such as age, smoking, BMI, hypertension, diabetes, dyslipidemia, and* HRC* S96A polymorphism (Table 4).

Given the large percentage of hypertensive, dyslipidemic, and diabetic subjects in our cohort, we conducted a stratified association analysis of the genetic variants in the presence or absence of these comorbidities.

Also in the subgroups, Hardy-Weinberg equilibrium was not reached for the* RyR2* Q2958R variant.

The distribution of* RyR2* Q2958R genotypes was not significantly different in dyslipidemic and nondyslipidemic patients between the two groups (Table 5). The same is true in diabetic and nondiabetic individuals, as we believe that the difference apparently emerging is exclusively linked to the low number of patients with diabetes. On the contrary, in hypertensive subjects we found a higher, statistically significant frequency of* RyR2* QQ genotype among VT/VF patients (*p* = 0.0001 with Fisher's exact test and *p* = 0.004 using Armitage trend test), which was associated with an increased risk of malignant ventricular arrhythmias (RR 2.51; IC 95% 1.96–3.23; *p* = 0.0001), whereas in hypertension-free patients the genotypic percentages were the same in the two groups (*p* = 1).

Additionally, in these subgroups, no significant association was observed between the* HRC* S96A genotypes and ventricular arrhythmias (data not shown).

When analyzed by allele status (Table 6), there were no significant differences in baseline clinical characteristics between* RyR2*QQ patients and QR or RR individuals; however, we observed an increase in the percentage of hypertensive subjects in the QQ genotype (66.7%) compared to the other genotypes (43.8%). This analysis indicates that the correlation between the 2958QQ genotype and the history of sustained VT/VF in our patients is independent of other clinical characteristics.

## 4. Discussion

In the present study we found that the* RyR2*QQ genotype seems to be associated with a strong trend towards increased susceptibility to life-threatening arrhythmias in patients receiving ICD therapy for primary and secondary prevention. The association was significant in the general population (*p* = 0.004) and was more evident in hypertensive patients (*p* = 0.0001). A marked HWD in the genotypic distribution of Q2958R polymorphism for* RyR2* gene was observed. Moreover, this SNP seems to be an independent risk factor for an increased risk of ventricular arrhythmias, as evidenced by multivariate analysis. Tiso et al. [13] described for the first time the Q2958R polymorphism in* RyR2* gene. As reported in dbSNP (http://www.ncbi.nlm.nih.gov/SNP/), the Q2958 allele frequency was estimated to be 72.1% in the European population (573 subjects) and 64% in the population of North America (120 subjects). In our cohort (335 subjects), the frequency is lowered to 53% with a heterozygous prevalence of 90.6%. Deviation from HWE may be due to biologic or nonbiologic reasons. Genotyping errors are one of the possible sources of HWD, but they are generally small and do not generate sufficient deviations from HWE to be detected. However, to rule out genotyping errors, we reanalyzed this polymorphism by the use of direct sequencing and in all cases sequencing data were consistent with AS-PCR results. To exclude a selection bias (population stratification) due to our inclusion and exclusion criteria, we enrolled and genotyped 100 healthy subjects, obtaining the same genotype distribution.

Another possible cause of deviation from HWE could be a differential survival of subjects with different genotypes: while the newborns of a population may be in HWE, the elderly individuals deviate from HWE. So we think that the prevalence of heterozygosity could be explained by a relatively benign effect of the QR condition on RyR2 channel function. However, this advantage is lacking in the homozygous QQ, becoming risk-conferring in the setting of imposed stress load due to hypertension.

In recent reports, it has been shown that defective interdomain interaction between the IP-domain (putative partner domain of I-domain, which remains to be identified) and the I-domain (amino acids 3722–4610) causes various proarrhythmic states, such as increased frequency of spontaneous Ca^2+^ sparks and the appearance of DAD [23]. We hypothesize that the presence of glutamic acid in position 2958 could weaken the IP-domain/I-domain interaction, keeping RyR2 in a slightly less closed form, resulting in greater Ca^2+^ release. In this context, stress factors such as hypertension can amplify the phenotypic effects of the Q2958 allele [24]. Furthermore, the Q2958R polymorphism lies within the proposed modulator region (MR1, residues 2618–3015) that has been highly conserved during ryanodine receptor evolution [14]. Three potential calmodulin-binding sites, from residues 2774–2806, 2876–2897, and 2997–3015, have been found in this region [25]. The presence of glutamic acid in this region could weaken the interaction of RyR2 with modulators especially in hypertensive patients.

Alternatively, this variant might be in linkage disequilibrium with a gene that contributes to disease susceptibility or affects survival, or with a gene that is associated with the choice of mates, justifying the Hardy-Weinberg disequilibrium.

Different from Arvanitis et al. [19] in our cohort, we did not find any association with the S96A polymorphism in the HRC gene and an increased risk of arrhythmias. The discordant results may be due to the different method of patient recruitment. Only patients with idiopathic dilated cardiomyopathy were analyzed in the work of Arvanitis et al. [19].

## 5. Conclusions

To date, our study is the first one to analyze the possible role of* RyR2* Q2958R polymorphism in SCD, showing that it might contribute to the onset of malignant cardiac arrhythmias. Stress load due to hypertension seems to modulate this effect. The association that we found may help to determine the arrhythmic risk in HF patients, who could benefit from an ICD implantation according to the ACC/AHA guidelines. The limit of our study is that the genotype distribution of the* RyR2* Q2958R variant showed deviation from HWE. This implies a selected rather than a random sample, invalidating direct comparisons with other populations.

However, we must keep in mind that single-nucleotide polymorphisms are only partial contributors to an individual's risk for developing a disease.

Therefore, our results should be regarded with caution and our findings regarding RyR2 polymorphism should be confirmed in future prospective larger-scale clinical trials specifically designed, comparing similar study groups in primary prevention with the same phenotype (i.e., underlying disease) and adequately powered to detect genotype-specific differences.

## Figures and Tables

**Table 1:** Primers sequences, annealing temperatures, and fragment size.

Gene	SNP	Allele	Primer sequence 5′→3′	Fragment size (bp)	*T*_*a*_
*RyR2*	Q2958R (A>G)	A	*Upper*: GGAGAACATTTCCCTTATGACC**A**	476	60°C
			*Lower*: GCAGGACTAAGGTCCCACAA		
		G	*Upper*: GAGAACATTTCCCTTATGACC**G**	476	60°C
			*Lower*: GCAGGACTAAGGTCCCACAA		

*HRC*	S96A (G>T)	G	*Upper*: AAGGAGGATGAAGATGCC**G**	347	62°C
			*Lower*: TCCTCTTCCTCCTCCTCCTC		
		T	*Upper*: AAAAGGAGGATGAAGATGCC**T**	347	62°C
			*Lower*: TCCTCTTCCTCCTCCTCCTC		

Bold letters in primer sequences highlight the allele-specific nucleotide, while underlined letters highlight mismatch nucleotide.

**Table 2:** Demographic data and clinical features of the study population according to arrhythmias occurrence.

	Total *n* = 235	Group I *n* = 101	Group II *n* = 134	*p* value
Demographic				
Male sex, *n* (%)	182 (77.4%)	86 (85.1%)	96 (71.6%)	0.0177
Age (years)	73 ± 8	73 ± 8	73 ± 8	1.0000
BMI (kg/m^2^)	27 ± 5	28 ± 5	27 ± 4	0.0887
Current smoking, *n* (%)	122 (51.5%)	53 (52.5%)	69 (50.7%)	0.5958
HF etiology				
IDCM, *n* (%)	83 (35.3%)	19 (18.8%)	64 (47.8%)	0.0001
Dilated IHD, *n* (%)	108 (46.0%)	42 (41.6%)	66 (49.2%)	
Nondilated IHD, *n* (%)	29 (12.3%)	29 (28.7%)	0 (0%)	
Other HD, *n* (%)	15 (6.4%)	11 (10.9%)	4 (3.0%)	
CRT, *n* (%)	64 (27.2%)	18 (17.8%)	46 (34.3%)	0.0051
NYHA class, *n* (%)				
I	34 (14.5%)	16 (15.8%)	18 (13.4%)	0.4548
II	106 (45.1%)	50 (49.5%)	56 (41.8%)	
III	92 (39.1%)	34 (33.7%)	58 (43.3%)	
IV	3 (1.3%)	1 (1%)	2 (1.5%)	
Comorbidities				
Hypertension, *n* (%)	107 (45.5%)	50 (49.5%)	57 (42.5%)	0.2936
Diabetes, *n* (%)	90 (38.3%)	40 (39.6%)	50 (37.3%)	0.7866
Dyslipidemia, *n* (%)	96 (40.8%)	42 (41.6%)	54 (40.3%)	0.8937
Atrial fibrillation, *n* (%)	45 (19.1%)	15 (14.9%)	30 (22.4%)	0.1807
Echo				
LVEDD, (mm)	59 ± 10	60 ± 12	57 ± 13	0.0716
LVESD, (mm)	47 ± 9	48 ± 8	46 ± 10	0.1002
LVEF, (%)	33 ± 10	32 ± 9	34 ± 11	0.1377
Drug therapy				
Beta-blockers, *n* (%)	200 (85.1%)	86 (85.1%)	114 (85.1%)	1.0000
ACE-inhibitors or ARB, *n* (%)	184 (78.3%)	82 (81.2%)	102 (76.1%)	0.4247
Antialdosterone, *n* (%)	150 (63.8%)	61 (60.3%)	89 (66.4%)	0.4107
Diuretics, *n* (%)	210 (89.4%)	85 (84.1%)	123 (93.3%)	0.0971
Amiodarone, *n* (%)	95 (40.4%)	61 (60.4%)	34 (25.4%)	0.0001

ARB: angiotensin receptor blockers; BMI: body mass index; HD: heart disease; HF: heart failure; IDCM: idiopathic dilated cardiomyopathy; IHD: ischemic heart disease; CRT: cardiac resynchronization therapy; LVEDD: left ventricular end-diastolic diameter; LVEF: left ventricular ejection fraction; LVESD: left ventricular end-systolic diameter.

**Table 3:** Genotypic frequency of the two analyzed polymorphisms in the study population and according to arrhythmias occurrence.

	Total	Group I	Group II	*p* value
*RyR2* Q2958R (A>G) (*n* = 235)				
QQ	18 (7.7%)	14 (13.9%)	4 (3.0%)	0.0040^*∗*^
QR	213 (90.6%)	85 (84.2%)	128 (95.5%)	
RR	4 (1.7%)	2 (1.9%)	2 (1.5%)	
*HRC* S96A (G>T) (*n* = 235)				
SS	38 (16.2%)	12 (11.9%)	26 (19.4%)	0.1750
SA	145 (61.7%)	69 (68.3%)	76 (56.7%)	
AA	52 (22.1%)	20 (19.8%)	32 (23.9%)	

*∗* with Fisher's exact test, while *p* = 0.0086 using Armitage trend test.

**Table 4:** Multiple logistic regression analysis.

	Odds ratio	95% CI	*p* value

Age	0.9983	0.9643 to 1.0335	0.9235
BMI	0.9926	0.5558 to 1.7724	0.9799
Diabetes	0.9320	0.5275 to 1.6468	0.8085
Dyslipidemia	0.8714	0.4977 to 1.5257	0.6300
Hypertension	0.7225	0.4106 to 1.2715	0.2597
Smoking	0.9118	0.5100 to 1.6302	0.7554
*HRC*	0.6929	0.4720 to 1.0173	0.0612
*RyR2*	2.5559	1.1394 to 5.7334	0.0228

BMI: body mass index.

**Table 5:** Genotypic frequency of* RyR2* Q2958R in the study population subgroups according to arrhythmias occurrence.

	Group I	Group II	*p* value
Hypertensive patients (*n* = 107)			
QQ	12 (24.0%)	0 (0%)	0.0001^*∗*^
QR	37 (74.0%)	56 (98.2%)	
RR	1 (2.0%)	1 (1.8%)	
Not hypertensive patients (*n* = 118)			
QQ	2 (4.3%)	3 (4.2%)	1
QR	43 (93.5%)	68 (94.4%)	
RR	1 (2.2%)	1 (1.4%)	
Dyslipidemic patients (*n* = 96)			
QQ	5 (11.9%)	6 (11.1%)	1
QR	36 (85.7%)	47 (87.0%)	
RR	1 (2.4%)	1 (1.9%)	
Not dyslipidemic patients (*n* = 129)			
QQ	4 (7.4%)	3 (4.0%)	0.6777
QR	49 (90.8%)	71 (94.7%)	
RR	1 (1.8%)	1 (1.3%)	
Diabetic patients (*n* = 90)			
QQ	6 (15.0%)	2 (4.0%)	0.0696
QR	33 (82.5%)	48 (96.0%)	
RR	1 (2.5%)	0 (0%)	
Not diabetic patients (*n* = 137)			
QQ	8 (14.3%)	2 (2.5%)	0.0204
QR	47 (83.9%)	77 (95.0%)	
RR	1 (1.8%)	2 (2.5%)	

*∗* with Fisher's exact test, while *p* = 0.0004 using Armitage trend test.

**Table 6:** Clinical characteristics and events stratified according to *RyR2* allele status.

	*RyR2* QQ *n* = 18	*RyR2* QR + RR *n* = 217	*p* value
Demographic			
Male sex, *n* (%)	13 (72.2%)	170 (78.3%)	0.5581
Age (years)	74 ± 7	72 ± 6	0.1811
BMI (kg/m^2^)	28 ± 6	28 ± 3	1.0000
Current smoking, *n* (%)	7 (38.9%)	98 (45.2%)	0.6330
HF etiology			0.6598
IDCM, *n* (%)	5 (27.8%)	76 (35.0%)	
Dilated IHD, *n* (%)	8 (44.5%)	100 (46.1%)	
Nondilated IHD, *n* (%)	3 (16.6%)	28 (12.9%)	
Other HD, *n* (%)	2 (11.1%)	13 (6.0%)	
NYHA class, *n* (%)			0.8741
I	3 (16.7%)	30 (13.8%)	
II	9 (50.0%)	98 (45.2%)	
III	6 (33.3%)	86 (39.6%)	
IV	0	3 (1.4%)	
VT/VF, *n* (%)	14 (77.8%)	87 (40.1%)	0.0025
Comorbidities			
Hypertension, *n* (%)	12 (66.7%)	95 (43.8%)	0.0837
Diabetes, *n* (%)	8 (44.4%)	82 (37.8%)	0.6187
Dyslipidemia, *n* (%)	11 (61.1%)	85 (39.2%)	0.0827
Echo			
LVEDD, (mm)	58 ± 10	61 ± 8	0.1354
LVESD, (mm)	47 ± 8	48 ± 6	0.5093
LVEF (%)	35 ± 10	32 ± 11	0.2643

BMI: body mass index; HD: heart disease; HF: heart failure; IDCM: idiopathic dilated cardiomyopathy; IHD: ischemic heart disease; LVEDD: left ventricular end-diastolic diameter; LVEF: left ventricular ejection fraction; LVESD: left ventricular end-systolic diameter.
